# Knowledge of Blood‐Borne Infections Among Secondary School Students in Riyadh, Saudi Arabia

**DOI:** 10.1002/hsr2.73283

**Published:** 2026-09-23

**Authors:** Fatimah Alabdulkareem, Homood A. Alharbi, Faten Helmy

**Affiliations:** ^1^ King Fahad Medical City Riyadh Saudi Arabia; ^2^ College of Nursing King Saud University Riyadh Saudi Arabia

**Keywords:** adolescents, blood‐borne infections, health education, Saudi Arabia, school health, secondary school students

## Abstract

**Background:**

Blood‐borne infections remain a major global public health concern. Adolescents may encounter preventable exposure risks, making adequate knowledge of transmission and prevention important. This study assessed knowledge of blood‐borne infections and associated sociodemographic factors and explored the association between previous exposure to health education and knowledge level among secondary school students in Riyadh, Saudi Arabia.

**Methods:**

An analytical cross‐sectional study was conducted among 686 students selected through multistage random sampling from four governmental secondary schools during the 2017–2018 academic year. Data were collected using a structured, self‐administered, expert‐reviewed questionnaire that demonstrated excellent internal consistency reliability (Cronbach's alpha = 0.954). Descriptive analyses, *χ* ^2^ tests, and multivariable multinomial logistic regression were performed. An exploratory *χ* ^2^ analysis examined the association between previous exposure to health education and knowledge level, with Cramer's V used to estimate effect size.

**Results:**

The response rate was 98%. Overall, 63.7% of participants had poor knowledge, 24.1% had moderate knowledge, and 12.2% had good knowledge. In bivariate analyses, knowledge level was significantly associated with gender, age, educational grade, educational track, and parental educational level. Previous exposure to health education was also significantly associated with knowledge level (*χ* ^2^(2) = 21.10, *p *< 0.001; Cramer's *V* = 0.175). After adjustment, male students had lower odds of moderate and good knowledge than female students. Selected categories of age, educational grade, and parental education also remained independently associated with knowledge level, whereas educational track was no longer independently associated.

**Implications for School Health Policy and Practice:**

The identified knowledge gaps may help inform age‐appropriate and culturally relevant school health education programs addressing the transmission and prevention of blood‐borne infections.

**Conclusion:**

Substantial knowledge gaps regarding blood‐borne infections were identified among secondary school students. These findings provide baseline evidence for future research and school health education initiatives. Contemporary studies are needed to determine whether similar knowledge gaps persist among secondary school students in Saudi Arabia.

## 1 Introduction

Blood‐borne infections, including hepatitis B virus (HBV), hepatitis C virus (HCV), and human immunodeficiency virus (HIV), remain major global public health concerns and contribute substantially to morbidity and mortality worldwide. In 2024, an estimated 240 million people were living with chronic HBV infection and 47 million with chronic HCV infection. In 2025, approximately 41.0 million people were living with HIV globally [1, 2, 3]. The World Health Organization continues to identify viral hepatitis as a major global public health challenge [1, 2].

Health promotion is a core component of disease prevention and involves enabling individuals and communities to increase control over and improve their health. Health literacy, including the ability to access, understand, and use health information, underpins this process [4]. For blood‐borne infections specifically, knowledge of transmission routes and preventive measures supports informed health‐related decisions and preventive practices.

Health education and disease prevention efforts increasingly focus on adolescents. Potential routes of exposure to blood‐borne infections include contact with infected blood, unsafe injections, sharing contaminated needles or sharp instruments, tattooing or piercing with inadequately sterilized equipment, and other practices involving exposure to infected blood or body fluids [1, 2]. Limited knowledge regarding transmission and prevention may contribute to misconceptions and potentially unsafe health‐related practices among adolescents.

Saudi Arabia has a large population of children and young people enrolled in educational institutions, providing a substantial school‐aged population in which health knowledge can be examined [5, 6]. During secondary school, adolescents increasingly encounter and interpret health information on their own, making schools a natural setting for supporting health literacy and preventive health knowledge.

The Health Sector Transformation Program under Saudi Vision 2030 emphasizes protection from health threats, reduction of communicable diseases, and health promotion as key components of national health‐system transformation [7]. Although previous Saudi studies have examined awareness and misconceptions regarding blood‐borne infections among specific population groups, including university students [8], evidence regarding knowledge among secondary school students remains limited.

Therefore, this study aimed to assess the level of knowledge regarding blood‐borne infections, including their transmission, prevention, and control, among secondary school students in Riyadh, Saudi Arabia; examine the sociodemographic factors associated with knowledge level; and explore the association between previous exposure to health education and knowledge level.

This study was intended to answer the following research questions:

1. What are the sociodemographic characteristics of secondary school students?
2. What is the level of knowledge regarding blood‐borne infections among secondary school students?
3. Is there an association between students' knowledge regarding blood‐borne infections and their sociodemographic characteristics?

Exploratory research question: Is previous exposure to health education associated with knowledge level regarding blood‐borne infections among secondary school students?

## 2 Methods

### 2.1 Participants

An analytical cross‐sectional study was conducted among secondary school students enrolled in governmental schools in the North sector of Riyadh, Saudi Arabia, during the 2017–2018 academic year. Riyadh is administratively divided into five main educational sectors (North, South, East, West, and Central). The North sector was randomly selected as the study setting. At the time of data collection, the North sector included 28 governmental secondary schools (20 female schools and 8 male schools).

A multistage random sampling technique was used. Initially, four government secondary schools were randomly selected, comprising two schools for male students and two schools for female students. Subsequently, 24 classes were randomly selected from the participating schools, with six classes selected from each school. In each school, two classes were randomly selected from each secondary school grade. The educational track was recorded according to the students' reported area of study.

All students in the selected classes who met the inclusion criteria were invited to participate. Inclusion criteria included male and female secondary school students from all educational grades and academic tracks, including both Saudi and non‐Saudi students.

The educational track was applicable only after students had chosen their area of study. Those who had not yet chosen between scientific and humanities tracks were classified as “General” in the dataset.

The population used to calculate the sample size comprised 1810 secondary school students enrolled in the selected schools during the 2017–2018 academic year. The minimum sample size was determined using Stephen K. Thompson's sample size formula for finite populations: *n *= *Np*(1 − *p*)/[(*N* − 1)(*d* ^2^/*Z* ^2^) + *p*(1 − *p*)], where *N* denotes the population size, *Z* is the *Z*‐value for the selected confidence level, *d* is the margin of error, and *p* is the estimated population proportion. A population size of 1810, a *Z*‐value of 1.96, a margin of error of 5% (*d* = 0.05), and an estimated population proportion of 0.50 (*p* = 0.50) yielded a minimum sample size of 317 participants. Since the finite population size (*N* = 1810) was used directly in the formula, the finite population correction was automatically applied.

A total of 700 eligible students from the selected classes were invited to participate, of whom 686 completed and returned the questionnaire, corresponding to a 98% response rate. No separate adjustment for anticipated nonresponse was applied to the minimum sample‐size calculation. The achieved sample of 686 exceeded the minimum required sample size of 317 participants. All 686 completed questionnaires were included in the final analysis.

### 2.2 Instrumentation

Data were collected using a structured, self‐administered questionnaire developed to assess secondary school students' knowledge regarding blood‐borne infections, including their prevention and control. The questionnaire was developed by the researchers based on a review of relevant literature [9, 10, 11, 12, 13].

The questionnaire consisted of three main sections:

- Sociodemographic characteristics, including age, gender, marital status, educational grade, educational track, parental education, parental work status, family monthly income, family size, and living arrangement. Marital status was collected as part of the sociodemographic data; however, because all participants were unmarried, this variable was excluded from the sociodemographic tables and subsequent analyses.
- Previous exposure to health education, including attendance at lectures or seminars related to blood‐borne infections, sexually transmitted diseases (STDs), HIV/AIDS, infectious diseases, blood donation/transfusion, and hepatitis.
- Knowledge regarding blood‐borne infections, including prevention and control. For descriptive purposes, the knowledge items covered four broad content categories: definitions and types of blood‐borne infections; modes of transmission; symptoms, complications, and treatment; and preventive measures. For domain‐level reliability analysis, these items were further organized into eight scoring domains: definitions and examples, microbe type, transmission modes, signs and symptoms, complications, incorrect practices, treatment, and prevention.

The questionnaire was administered to all participants in Arabic. The English version provided in Supporting Information S1 was prepared for reporting and publication purposes and was not administered to study participants.

The knowledge section contained 60 numbered entries (items 43–102); however, item 65 (“Other, please specify”) was an open‐ended, non‐scored item. Therefore, 59 items contributed to the total knowledge score. Each correct response received 1 point, whereas incorrect and “I do not know” responses received 0 points, resulting in a total score ranging from 0 to 59. Knowledge level was categorized using cut‐offs specified a priori in the original study protocol and not derived from the observed distribution of the study data: poor knowledge, <60% (0–35 points); moderate knowledge, 60% to <70% (36–41 points); and good knowledge, ≥70% (42–59 points). The full questionnaire and scoring system are provided in Supporting Information S1.

Content validity was determined by a group of six experts who examined the questionnaire for relevance, clarity, and appropriateness and recommended any necessary changes. A numerical Content Validity Index (CVI) was not calculated during the original assessment.

Item‐to‐total correlations were examined in the final analytical sample (*N* = 686) using Pearson's correlation coefficient. The correlations between individual knowledge items and the total knowledge score ranged from −0.021 to 0.657. Most correlations were statistically significant at *p *< 0.01 or *p *< 0.05; however, items 43 (*r* = 0.057, *p *= 0.139) and 92 (*r *= −0.021, *p *= 0.588) were not significantly correlated with the total knowledge score.

Items 43 and 92 were retained to preserve the questionnaire's content coverage and original scoring framework despite their non‐significant item‐to‐total correlations. Their results were therefore interpreted cautiously.

The questionnaire's reliability and feasibility were assessed in a pilot study involving 47 students from two schools that were excluded from the main study. The 59 scored knowledge items demonstrated excellent internal consistency reliability (Cronbach's alpha = 0.954). The domain‐level Cronbach's alpha coefficients were 0.819 for definitions and examples, 0.784 for microbe type, 0.855 for the modes of transmission, 0.736 for signs and symptoms, 0.786 for complications, 0.899 for incorrect practices, 0.629 for treatment, and 0.928 for prevention. The comparatively lower reliability of the treatment domain (*α *= 0.629) was interpreted cautiously, particularly because this domain contained only three items. The pilot study also assessed the questionnaire's feasibility, clarity, organization, and estimated completion time. Based on the pilot findings, minor modifications were made to improve wording clarity and question organization. The pilot participants were not included in the final analytical sample of 686 students. The full questionnaire and scoring system are provided in Supporting Information S1, and complete item‐level results are presented in Supporting Information S2: Table S1.

### 2.3 Procedure

Data collection was conducted over a 4‐week period in 2018, following Institutional Review Board approval. Prior to distributing the questionnaire, the participants received information regarding the purpose of the study and how to complete the questionnaire. The researcher distributed the questionnaires during free periods, and participants took approximately 20 min to complete them.

The principal researcher collected data from female students while a trained male colleague collected data from male students. A total of 686 completed questionnaires were returned, resulting in an overall response rate of 98%.

Ethical approval was obtained from the Institutional Review Board (IRB) of King Khaled University Hospital, Riyadh, Saudi Arabia (Approval No. E‐18‐3425), prior to data collection.

Written informed consent was obtained in accordance with the ethical requirements and procedures approved by the Institutional Review Board. Participation was voluntary, and participants were informed that they could withdraw from the study at any time without providing a reason. The study was conducted in accordance with the ethical principles for medical research involving human subjects of King Saud University.

Clinical trial registration was not applicable to this study.

### 2.4 Data Analysis

The original descriptive, bivariate, and multinomial logistic regression analyses were conducted using IBM SPSS Statistics, version 22. Additional model diagnostics and exploratory analyses, including VIF and tolerance calculations, model‐fit indices, Holm‐adjusted *p* values, and analyses of previous health education exposure, were conducted using Python, version 3.12.

All 686 completed questionnaires were included in the analysis. No missing data were identified for the variables included in the primary analyses. Blank responses were limited to optional open‐ended “Other, please specify” fields, which were not included in the knowledge score.

Descriptive statistics, including frequencies, percentages, means, and standard deviations, were used to describe participant characteristics and knowledge scores. Item‐to‐total correlations were examined using Pearson's correlation coefficient. The internal consistency reliability of the overall knowledge scale and its domains was assessed using Cronbach's alpha coefficient.

Pearson's *χ* ^2^ test was used to examine associations between overall knowledge level (poor, moderate, and good) and sociodemographic characteristics. When the assumptions of Pearson's chi‐square test were not met because of small expected cell counts, Monte Carlo‐estimated *p* values based on 200,000 samples were reported. Cramer's *V* was calculated as a measure of effect size. For inferential analyses, parental educational level was collapsed into five categories: illiterate/read and write, elementary school, intermediate school, secondary school, and diploma/university education or above.

An exploratory analysis was conducted to examine the association between previous exposure to health education and knowledge level. A composite variable representing any previous health education exposure was coded as “yes” when a participant reported attending at least one lecture or seminar related to blood‐borne infections, infectious diseases, sexually transmitted diseases, hepatitis, HIV/AIDS, or blood donation/transfusion. Its association with knowledge level was examined using Pearson's *χ* ^2^ test, and Cramer's *V* was calculated as an effect‐size measure. Associations for the six individual education topics were also examined separately, with Holm‐adjusted *p* values used to account for multiple comparisons. The composite exposure comparison was not included in the Holm adjustment. These exposure variables were not added to the multivariable model because the analyses were exploratory and the inclusion of additional parameters would have increased the potential for model overfitting.

To identify sociodemographic factors independently associated with knowledge level, a multivariable multinomial logistic regression analysis was performed using poor knowledge as the reference outcome category. Age group, gender, nationality, educational grade, educational track, mother's educational level, and father's educational level were entered simultaneously into the model. Adjusted odds ratios (aORs), 95% confidence intervals (CIs), and *p* values were reported.

Multicollinearity among the predictor variables was assessed using variance inflation factors (VIFs) and tolerance values. A VIF greater than five or a tolerance less than 0.20 was considered an indicator for potential multicollinearity. Model adequacy was evaluated using the likelihood‐ratio test, McFadden's pseudo‐R^2^, the Akaike Information Criterion (AIC), and the Bayesian Information Criterion (BIC). The number of observations in the smallest outcome category relative to the number of estimated predictor coefficients was also considered when interpreting model stability. All statistical tests were two‐tailed, and statistical significance was set at *p *< 0.05.

Efforts to reduce potential selection and information bias included multistage random sampling, inclusion of both male and female schools, use of an anonymous self‐administered questionnaire, and administration of a structured instrument that underwent expert review and demonstrated high internal consistency reliability. Nonetheless, self‐reporting and the restriction of the sample to governmental schools in the North Riyadh sector may have introduced residual bias and limited the generalizability of the findings.

This study was reported in accordance with the STROBE guidelines for cross‐sectional studies.

## 3 Results

### 3.1 Sociodemographic Characteristics

**Figure 1:**
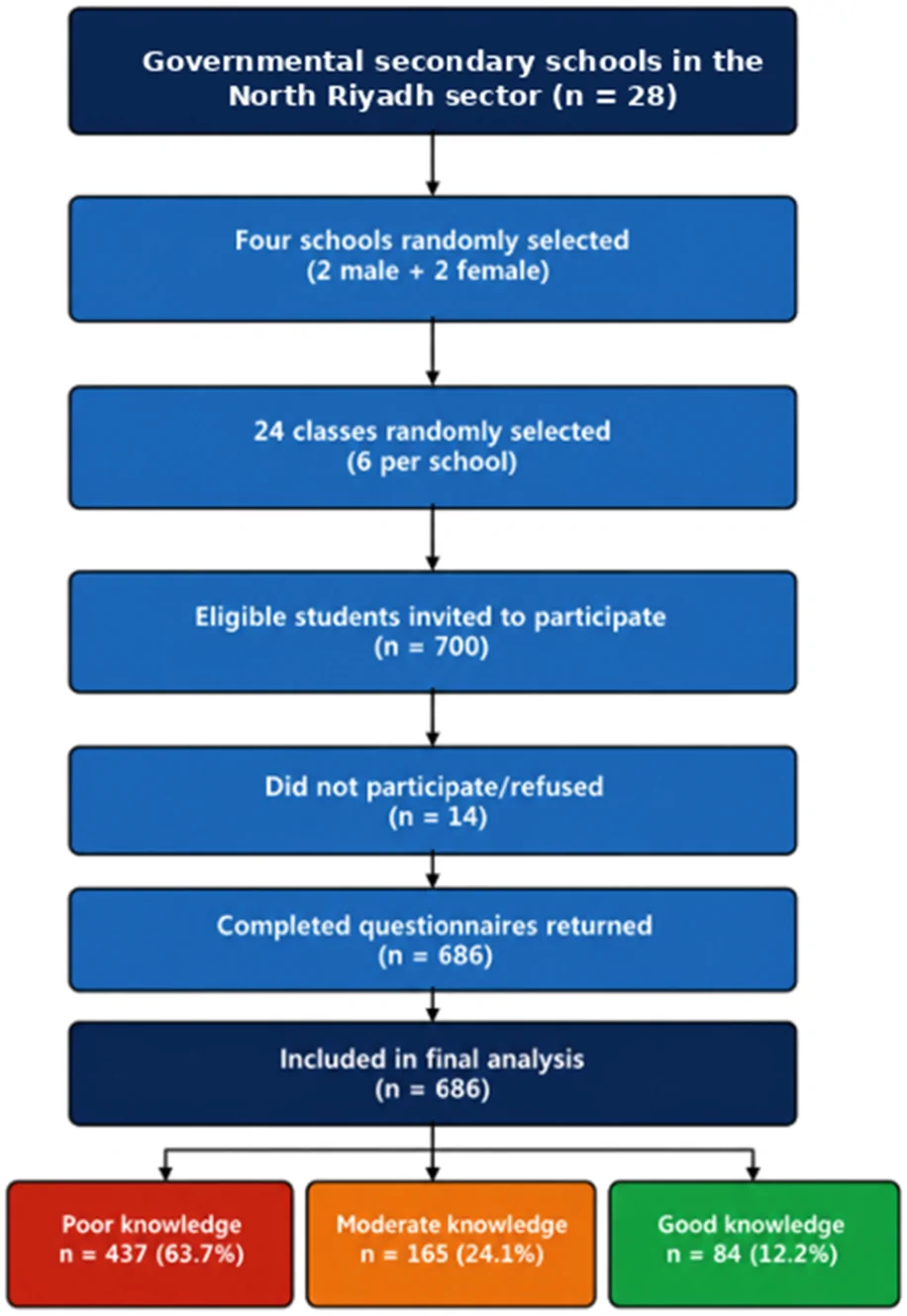
Participant flow diagram.

A total of 700 eligible students were invited to participate in the study; 686 completed and returned the questionnaire, representing a 98% response rate. All 686 completed questionnaires were included in the analysis. The sociodemographic characteristics of the participants are presented in Table 1. The average age of the participants was 16.97 years (SD = 1.01). Most participants were aged >16 to ≤18 years (62.83%), while 32.65% were aged ≤16 years and 4.52% were aged >18 years. Among the respondents, 53.64% were male and 46.36% were female. The majority of the participants were Saudi nationals (59.8%). Figure 1 shows the progression through each stage of the study.

**Table 1:** Sociodemographic characteristics of participants (*N* = 686).

Variable	Category	*n*	%
Age group	≤ 16 years	224	32.65
	> 16 to ≤ 18 years	431	62.83
	> 18 years	31	4.52
Gender	Male	368	53.64
	Female	318	46.36
Nationality	Saudi	410	59.77
	Non‐Saudi	276	40.23
Educational grade	Grade 1	216	31.49
	Grade 2	224	32.65
	Grade 3	246	35.86
Educational track	Scientific	327	47.67
	Humanities	138	20.12
	General	221	32.22
Number of family members	1–3	17	2.47
	4–6	249	36.29
	> 6	420	61.22
Living arrangement	Family	677	98.68
	Relatives	5	0.72
	Other places	4	0.58
Mother's educational level	Illiterate/read and write	76	11.08
	Elementary school	43	6.27
	Intermediate school	87	12.68
	Secondary school	226	32.94
	Diploma/university education or above	254	37.03
Father's educational level	Illiterate/read and write	45	6.56
	Elementary school	19	2.77
	Intermediate school	58	8.45
	Secondary school	160	23.32
	Diploma/university education or above	404	58.89
Mother's work status	Employed	153	22.30
	Not employed	506	73.76
	Retired	21	3.06
	Deceased	6	0.87
Father's work status	Employed	521	75.95
	Not employed	27	3.94
	Retired	104	15.16
	Deceased	34	4.96
Family monthly income	< 3000 SAR	49	7.14
	3000–6000 SAR	98	14.29
	6001–9000 SAR	61	8.89
	> 9000 SAR	204	29.74
	Do not know	274	39.94

Participants were relatively evenly distributed across the three secondary school grades: 31.5% were in Grade 1, 32.6% in Grade 2, and 35.9% in Grade 3. In terms of family characteristics, 61.2% of the participants reported having over six family members, and 98.7% lived with their families.

### 3.2 Previous Exposure to Health Education

Previous exposure to health education related to blood‐borne infections was limited. Among the 686 participants, 163 (23.76%) reported previous attendance at a lecture or seminar about HIV/AIDS, 140 (20.41%) about infectious diseases, 136 (19.85%) about blood donation/transfusion, 119 (17.35%) about blood‐borne infections, 92 (13.41%) about sexually transmitted diseases, and 59 (8.60%) about hepatitis.

Overall, 278 students (40.5%) reported attending at least one lecture or seminar related to the assessed health topics. Among students with any previous health education exposure, 53.6% had poor knowledge, 29.9% had moderate knowledge, and 16.5% had good knowledge, compared with 70.6%, 20.1%, and 9.3%, respectively, among those without previous exposure. Previous health education exposure was significantly associated with knowledge level (*χ* ^2^(2) = 21.10, *p *< 0.001; Cramer's *V* = 0.175). Each of the six individual education topics also remained significantly associated with knowledge level after Holm adjustment for multiple comparisons (adjusted *p* values ≤ 0.0035), with Cramer's V values ranging from 0.128 to 0.226. The largest effect size was observed for previous education related to sexually transmitted diseases (Cramer's *V *= 0.226). Complete results are presented in Supporting Information S3: Table S2.

### 3.3 Knowledge Regarding Blood‐Borne Infections

#### 3.3.1 Knowledge of Disease Concepts

A large majority of participants (80.17%) identified blood transfusion as a potential route of transmission of blood‐borne infections. More than half (56.71%) correctly identified viruses as causative agents. However, knowledge of selected symptoms was lower; 49.71% recognized fatigue and 41.40% recognized high fever as possible symptoms of blood‐borne infections.

#### 3.3.2 Knowledge of Transmission and Risk Factors

Awareness of selected transmission routes and high‐risk activities was relatively high. Sharing needles, syringes, or sharp instruments was identified as a risk factor by 86.01% of participants, sharing cupping equipment without disinfection by 76.09%, and sharing shaving blades by 66.33%.

#### 3.3.3 Overall Knowledge Scores

Overall knowledge regarding blood‐borne infections was low. Poor knowledge was observed among 63.7% of participants, while 24.1% demonstrated moderate knowledge and 12.2% demonstrated good knowledge.

#### 3.3.4 Factors Associated With Knowledge

Several sociodemographic characteristics were significantly associated with knowledge level in the bivariate analysis, including gender (*p *< 0.001), age (*p *= 0.004), educational grade (*p *< 0.001), educational track (*p *= 0.008), mother's educational level (*p *= 0.025), and father's educational level (*p *= 0.005). The associations between knowledge level and sociodemographic characteristics are presented in Table 2. Among the variables with statistically significant associations, Cramer's *V* values ranged from 0.101 to 0.203, with the largest effect size observed for gender (Cramer's *V* = 0.203).

**Table 2:** Bivariate associations between sociodemographic characteristics and knowledge level among participants (*N* = 686).

Sociodemographic variable	Category	Poor knowledge *n* (%)	Moderate knowledge *n* (%)	Good knowledge *n* (%)	*χ* ^2^	df	*p* value	Cramer's *V*
Gender	Female	173 (54.4)	87 (27.4)	58 (18.2)	28.136	2	< 0.001	0.203
	Male	264 (71.7)	78 (21.2)	26 (7.1)				
Age group	≤16 years	148 (66.1)	61 (27.2)	15 (6.7)	15.436	4	0.004	0.106
	>16 to ≤18 years	264 (61.3)	102 (23.7)	65 (15.1)				
	>18 years	25 (80.6)	2 (6.5)	4 (12.9)				
Nationality	Saudi	273 (66.6)	92 (22.4)	45 (11.0)	3.773	2	0.152	0.074
	Non‐Saudi	164 (59.4)	73 (26.4)	39 (14.1)				
Educational grade	Grade 1	150 (69.4)	53 (24.5)	13 (6.0)	24.903	4	< 0.001	0.135
	Grade 2	148 (66.1)	55 (24.6)	21 (9.4)				
	Grade 3	139 (56.5)	57 (23.2)	50 (20.3)				
Educational track	Scientific	197 (60.2)	76 (23.2)	54 (16.5)	13.924	4	0.008	0.101
	Humanities	88 (63.8)	33 (23.9)	17 (12.3)				
	General	152 (68.8)	56 (25.3)	13 (5.9)				
Number of family members	1–3	8 (47.1)	7 (41.2)	2 (11.8)	4.244	4	0.373^a^	0.056
	4–6	154 (61.8)	65 (26.1)	30 (12.0)				
	>6	275 (65.5)	93 (22.1)	52 (12.4)				
Living arrangement	Family	431 (63.7)	162 (23.9)	84 (12.4)	2.601	4	0.636^a^	0.044
	Relatives	4 (80.0)	1 (20.0)	0 (0.0)				
	Other places	2 (50.0)	2 (50.0)	0 (0.0)				
Mother's educational level	Illiterate/read and write	54 (71.1)	18 (23.7)	4 (5.3)	17.580	8	0.025	0.113
	Elementary school	21 (48.8)	15 (34.9)	7 (16.3)				
	Intermediate school	56 (64.4)	21 (24.1)	10 (11.5)				
	Secondary school	160 (70.8)	41 (18.1)	25 (11.1)				
	Diploma/university education or above	146 (57.5)	70 (27.6)	38 (15.0)				
Father's educational level	Illiterate/read and write	25 (55.6)	16 (35.6)	4 (8.9)	22.190	8	0.005	0.127
	Elementary school	15 (78.9)	1 (5.3)	3 (15.8)				
	Intermediate school	41 (70.7)	14 (24.1)	3 (5.2)				
	Secondary school	117 (73.1)	32 (20.0)	11 (6.9)				
	Diploma/university education or above	239 (59.2)	102 (25.2)	63 (15.6)				
Mother's work status	Employed	98 (64.1)	40 (26.1)	15 (9.8)	9.618	6	0.137^a^	0.084
	Not employed	326 (64.4)	119 (23.5)	61 (12.1)				
	Retired	11 (52.4)	4 (19.0)	6 (28.6)				
	Deceased	2 (33.3)	2 (33.3)	2 (33.3)				
Father's work status	Employed	329 (63.1)	126 (24.2)	66 (12.7)	3.096	6	0.797	0.048
	Not employed	18 (66.7)	8 (29.6)	1 (3.7)				
	Retired	70 (67.3)	22 (21.2)	12 (11.5)				
	Deceased	20 (58.8)	9 (26.5)	5 (14.7)				
Family monthly income	<3000 SAR	30 (61.2)	15 (30.6)	4 (8.2)	6.102	8	0.636	0.067
	3000–6000 SAR	60 (61.2)	25 (25.5)	13 (13.3)				
	6001–9000 SAR	40 (65.6)	13 (21.3)	8 (13.1)				
	>9000 SAR	128 (62.7)	44 (21.6)	32 (15.7)				
	Do not know	179 (65.3)	68 (24.8)	27 (9.9)				

*Note:* Percentages are calculated within each sociodemographic category (row percentages). *χ* ^2 ^= Pearson's *χ* ^2^ statistic; df = degrees of freedom. Cramer's V represents the effect‐size measure. Statistical significance was set at *p *< 0.05.

^a^ A Monte Carlo *p* value based on 200,000 samples was reported because the assumptions of Pearson's *χ* ^2^ test were not met due to small expected cell counts.

In the multivariable multinomial logistic regression analysis, age, gender, nationality, educational grade, educational track, mother's educational level, and father's educational level were entered simultaneously to examine their adjusted associations with knowledge level. The overall model was statistically significant (likelihood‐ratio *χ* ^2^(32) = 106.22, *p *< 0.001), with a McFadden's pseudo‐R^2^ of 0.087, an AIC of 1178.94, and a BIC of 1332.99. Predictor VIFs ranged from 1.08 to 6.94. The highest values were observed for Grade 1 (VIF = 6.94; tolerance = 0.14) and the general educational track (VIF = 5.69; tolerance = 0.18), reflecting their close relationship; all remaining VIFs were below 2.5. Because the good‐knowledge category included 84 participants and 16 predictor coefficients were estimated for each outcome comparison, estimates for this category were interpreted cautiously because of the potential for overfitting and instability. Compared with participants with poor knowledge, students aged >18 years had lower adjusted odds of moderate knowledge than those aged >16 to ≤18 years (aOR = 0.19, 0.04–0.84, *p *= 0.029). Male students had lower adjusted odds of moderate knowledge than female students (aOR = 0.58, 0.39–0.87, *p *= 0.008), as did students whose mothers had secondary education compared with those whose mothers had diploma/university education or above (aOR = 0.56, 0.35–0.89, *p *= 0.015).

For good versus poor knowledge, male students had lower adjusted odds than female students (aOR = 0.24, 0.14–0.43, *p *< 0.001). Grade 2 students had lower adjusted odds of good knowledge than Grade 3 students (aOR = 0.38, 0.20–0.69, *p *= 0.002). Students whose fathers had intermediate education (aOR = 0.26, 0.07–0.93, *p *= 0.038) or secondary education (aOR = 0.39, 0.18–0.84, *p *= 0.015) also had lower adjusted odds of good knowledge than those whose fathers had diploma/university education or above. The educational track was not independently associated with knowledge level after adjustment. The full results of the multivariable multinomial logistic regression analysis are presented in Table 3.

**Table 3:** Multivariable multinomial logistic regression analysis of factors associated with knowledge level (*N* = 686).

Predictor	aOR	95% CI	*p* value
Moderate knowledge vs. poor knowledge			
Age ≤16 vs. >16–≤18 years	1.24	0.69–2.25	0.468
Age >18 vs. >16–≤18 years	**0.19**	**0.04–0.84**	**0.029**
Male vs. female	**0.58**	**0.39–0.87**	**0.008**
Non‐Saudi vs. Saudi	1.14	0.76–1.70	0.525
Grade 1 vs. grade 3	0.49	0.17–1.40	0.182
Grade 2 vs. grade 3	0.79	0.49–1.26	0.324
General vs. scientific track	1.46	0.57–3.78	0.431
Humanities vs. scientific track	0.97	0.56–1.68	0.918
Mother: Elementary vs. diploma/university education or above	1.93	0.87–4.26	0.104
Mother: Illiterate/read and write vs. diploma/university education or above	0.83	0.40–1.70	0.610
Mother: Intermediate vs. diploma/university education or above	1.03	0.53–1.98	0.933
Mother: Secondary vs. diploma/university education or above	**0.56**	**0.35–0.89**	**0.015**
Father: Elementary vs. diploma/university education or above	0.19	0.02–1.49	0.113
Father: Illiterate/read and write vs. diploma/university education or above	1.65	0.75–3.61	0.211
Father: Intermediate vs. diploma/university education or above	0.77	0.37–1.58	0.469
Father: Secondary vs. diploma/university education or above	0.67	0.41–1.11	0.124
Good knowledge vs. poor knowledge			
Age ≤16 vs. >16–≤18 years	0.61	0.24–1.53	0.291
Age >18 vs. >16–≤18 years	0.59	0.18–1.95	0.389
Male vs. female	**0.24**	**0.14–0.43**	**<0.001**
Non‐Saudi vs. Saudi	1.03	0.60–1.76	0.925
Grade 1 vs. grade 3	0.55	0.10–2.94	0.488
Grade 2 vs. grade 3	**0.38**	**0.20–0.69**	**0.002**
General vs. scientific track	0.53	0.11–2.49	0.424
Humanities vs. scientific track	0.68	0.33–1.39	0.287
Mother: elementary vs. diploma/university education or above	1.61	0.57–4.59	0.371
Mother: illiterate/read and write vs. diploma/university education or above	0.46	0.14–1.53	0.205
Mother: intermediate vs. diploma/university education or above	1.13	0.46–2.76	0.783
Mother: secondary vs. diploma/university education or above	0.73	0.40–1.34	0.309
Father: elementary vs. diploma/university education or above	1.07	0.26–4.39	0.921
Father: illiterate/read and write vs. diploma/university education or above	0.91	0.27–3.07	0.873
Father: intermediate vs. diploma/university education or above	**0.26**	**0.07–0.93**	**0.038**
Father: secondary vs. diploma/university education or above	**0.39**	**0.18–0.84**	**0.015**

*Note:* Poor knowledge was the reference outcome category. Reference categories for the predictors were age >16 to ≤18 years, female gender, Saudi nationality, Grade 3, scientific track, and diploma/university education or above for mother's and father's educational level. Statistically significant values are shown in bold. The overall multivariable multinomial logistic regression model was statistically significant (likelihood‐ratio *χ* ^2^(32) = 106.22, *p *< 0.001).

Abbreviations: aOR = adjusted odds ratio, CI = confidence interval.

### 3.4 Summary of Key Findings

**Figure 2:**
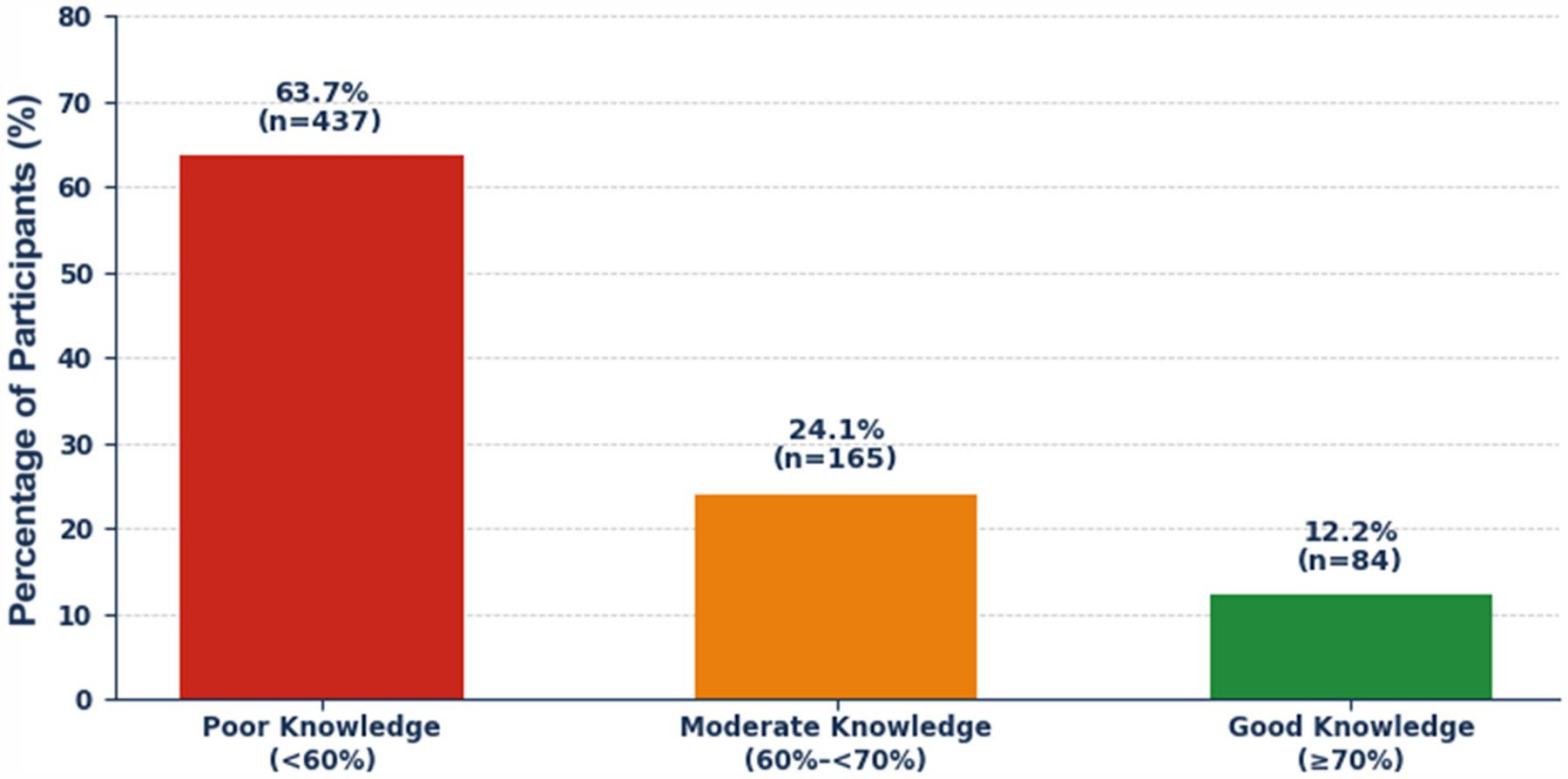
Distribution of overall knowledge levels regarding blood‐borne infections among participants (*N* = 686).

In summary, 63.7% of participants had poor knowledge of blood‐borne infections, 24.1% had moderate knowledge, and 12.2% had good knowledge. Participants demonstrated greater awareness of selected transmission routes and risk factors, such as sharing needles or sharp instruments, blood transfusion, and sharing cupping equipment without proper disinfection, while lower proportions correctly identified selected signs and symptoms and disease‐related concepts. In the bivariate analysis, knowledge level was significantly associated with gender, age, educational grade, educational track, and mother's and father's educational level. In an exploratory analysis, previous exposure to health education was also significantly associated with knowledge level; exposed students had a lower proportion of poor knowledge and a higher proportion of good knowledge than those without previous exposure. In the adjusted multinomial logistic regression analysis, gender, selected age and educational‐grade categories, and selected categories of mother's and father's educational level remained independently associated with knowledge level, whereas educational track was not independently associated after adjustment (Figure 2; Tables 2, 3, 4; Supporting Information S3: Table S2).

**Table 4:** Selected knowledge items regarding blood‐borne infections among participants (*N* = 686).

Knowledge item	Correct response, *n* (%)
Blood transfusion identified as a transmission route	550 (80.17)
Viruses identified as causative agents	389 (56.71)
Fatigue recognized as a symptom	341 (49.71)
High fever recognized as a symptom	284 (41.40)
Sharing needles, syringes, or sharp instruments identified as a risk factor	590 (86.01)
Sharing cupping instruments without disinfection identified as a risk factor	522 (76.09)
Sharing shaving blades identified as a risk factor	455 (66.33)

*Note:* The items presented in this table were selected to provide concise examples from the major knowledge domains discussed in the Results. Complete item‐level results for all 59 scored knowledge items are provided in Supporting Information S2: Table S1. Correct responses reflect the original study scoring key. Items addressing body fluids and breastfeeding should be interpreted cautiously because transmission risk and breastfeeding recommendations vary according to the specific blood‐borne infection.

## 4 Discussion

Significant gaps in knowledge about blood‐borne infections were identified among secondary school students in Riyadh, Saudi Arabia. Although participants were familiar with some transmission routes and high‐risk behaviors, overall knowledge was low, with nearly two‐thirds classified as having poor knowledge. These findings point to specific gaps in adolescents' health literacy related to blood‐borne infections that could be addressed through school‐based health education.

Participants showed strong familiarity with transmission routes such as blood transfusions, needle sharing, and unsterilized equipment, but other aspects of the disease, including causative agents and certain symptoms of blood‐borne infection, were less well understood. This suggests that students' knowledge of blood‐borne infections was partial rather than absent. Baig et al. reported misconceptions among university students in Saudi Arabia regarding blood donation and infections such as HIV, HBV, and HCV [8]. Taken together, these findings suggest that young people's knowledge of blood‐borne infection risks may be uneven, supporting a comprehensive rather than fragmented approach to health education.

Recent literature supports the importance of adolescent health literacy. In a scoping review of adolescents and young adults in the Eastern Mediterranean region, Sarhan et al. found considerable variation in health literacy and noted that evidence concerning this population in the region remains limited [14]. Alhodaib assessed e‐health literacy among secondary school students in Saudi Arabia, including their ability to locate, evaluate, and appropriately use health information from digital sources [15]. These studies place the knowledge gaps identified in the present study within a broader and more contemporary body of evidence.

Exposure to formal health education related to blood‐borne infections was limited, with fewer than half of the students reporting attendance at any relevant lecture or seminar. Nevertheless, previous exposure to health education was significantly associated with knowledge level. Students who reported attending at least one relevant activity had a lower proportion of poor knowledge and a higher proportion of good knowledge than those without previous exposure. Associations remained statistically significant for all six education topics after adjustment for multiple comparisons, with the largest effect size observed for education related to sexually transmitted diseases. These findings suggest that exposure to relevant health information may be associated with better knowledge of blood‐borne infections and highlight the potential value of structured school‐based health education. However, because the analyses were exploratory and cross‐sectional, they cannot establish that previous health education caused the observed differences. Students who attended these activities may have differed from non‐attendees in age, educational grade, personal interest, or access to health information, and the associations were not adjusted for these potential confounders. Therefore, prospective studies are needed to evaluate the effectiveness of structured educational interventions.

Several sociodemographic characteristics were associated with knowledge level in the bivariate analysis, including gender, age, educational grade, educational track, and mother's and father's educational level. However, after adjusting for other sociodemographic factors, the results of multivariable multinomial logistic regression analysis indicated that not all of the above factors were individually associated with the level of knowledge. According to our findings, gender and some categories of age and educational grade, as well as the educational level of parents, remained independently associated with moderate or good knowledge compared with poor knowledge, whereas educational track was no longer independently associated with knowledge level. These findings suggest that differences in knowledge may reflect multiple interrelated sociodemographic factors rather than the independent influence of any single characteristic. Given the cross‐sectional design, these associations should be interpreted cautiously and should not be considered evidence of causal relationships.

This study provides baseline data on knowledge of blood‐borne infections among secondary school students in Riyadh, a population whose health knowledge and preventive behaviors are still developing during adolescence. The findings may help identify priority topics for future school health education initiatives, particularly knowledge gaps regarding transmission, causative agents, and signs and symptoms, which should be addressed through evidence‐based, age‐appropriate, and culturally relevant approaches.

Although data collection was completed in 2018, HBV, HCV, and HIV remain significant global public health concerns [1, 2, 3]. The findings should therefore be interpreted within the period in which the data were collected. The study nonetheless offers baseline evidence on knowledge of blood‐borne infections among secondary school students in Riyadh, and further contemporary studies are needed to determine whether these knowledge gaps persist among secondary school students in Saudi Arabia.

Schools are a potentially valuable setting for developing adolescent health literacy. A systematic review by Smith et al. examined school‐based health‐related interventions among disadvantaged adolescents, noting the potential role of school settings in health literacy development, alongside considerable heterogeneity in intervention approaches and outcomes [16]. More recently, Khanal et al. reported improvements in adolescent health literacy and intentions to undertake health‐promoting actions following a multicomponent school‐based intervention, providing contemporary evidence supporting further evaluation of structured school‐based approaches in different settings [17].

### 4.1 Implications for School Health Policy, Practice, and Equity

The findings identify several areas that could be prioritized in future school‐based health education, including transmission routes, preventive measures, signs and symptoms, complications, treatment, and high‐risk behaviors. Age‐appropriate and culturally relevant educational approaches that consider sociodemographic differences and involve school nurses, teachers, and health educators may provide opportunities to improve access to reliable health information. However, because the present study did not evaluate an educational intervention, the effectiveness of such programs cannot be inferred from these findings and should be assessed in future intervention studies. Strengthening the evidence base for adolescent school health education may help inform future school health policies and initiatives aimed at improving health literacy and addressing knowledge gaps.

### 4.2 Limitations

This study has several limitations. First, the cross‐sectional design assessed students' knowledge and sociodemographic characteristics at a single point in time; therefore, temporal or causal relationships between variables cannot be established. Second, the multistage sampling design resulted in clustering of students within classes and schools, but the statistical analyses did not account for this clustering. Consequently, standard errors may have been underestimated, and the statistical significance of some associations may have been overstated. Third, data were collected using a self‐administered questionnaire and may have been affected by recall bias, misunderstanding of questionnaire items, or socially desirable responses. Fourth, the study was conducted in governmental secondary schools within only one educational sector in Riyadh, which limits the generalizability of the findings to other geographical areas, educational sectors, and private schools.

In addition, the close relationship between educational grade and educational track, particularly between Grade 1 and the general track, resulted in elevated VIFs for these predictors. The relatively small good‐knowledge category compared with the number of model parameters may also have increased the risk of overfitting and contributed to wide confidence intervals and unstable estimates. Therefore, the multivariable findings should be interpreted cautiously and require confirmation in larger samples. Although the knowledge‐level cut‐offs were specified a priori, they have not been externally validated and may have influenced the proportion of participants classified within each knowledge category.

Finally, because the data were collected in 2018, subsequent changes in school health programs, access to health information, and students' information‐seeking practices may limit the applicability of the findings to the current context. The findings should therefore be regarded as historical baseline evidence rather than an estimate of students' current knowledge. Contemporary studies are needed to determine whether the identified knowledge gaps persist.

## 5 Conclusion

This study identified significant knowledge gaps about blood‐borne infections among secondary school students in Riyadh, Saudi Arabia. Almost two‐thirds of students had poor overall knowledge. After adjustment for the included sociodemographic variables, gender and selected categories of age, educational grade, and parental education remained independently associated with knowledge level, whereas educational track was no longer independently associated after adjustment. These findings provide baseline evidence for future studies and the development of age‐appropriate school health education programs. Since the data were collected in 2018, more recent studies are needed to assess whether similar knowledge gaps persist among secondary school students in Saudi Arabia.

## Author Contributions

**Fatimah Alabdulkareem:** writing – original draft, conceptualization, methodology, investigation, formal analysis, data curation. **Homood A. Alharbi:** methodology, supervision, validation, writing – review and editing. **Faten Helmy:** formal analysis, validation, writing – review and editing, supervision.

## Funding

The authors have nothing to report.

## Ethics Statement

This study was approved by the Institutional Review Board of King Khaled University Hospital, Riyadh, Saudi Arabia (Approval No. E‐18‐3425).

## Consent

Written informed consent was obtained in accordance with the ethical requirements and procedures approved by the Institutional Review Board. Participation was voluntary, and participants were informed that they could withdraw from the study at any time without providing a reason. Confidentiality was maintained throughout data collection, entry, and analysis.

## Conflicts of Interest

The authors declare no conflicts of interest.

## Transparency Statement

The corresponding author affirms that this manuscript is an honest, accurate, and transparent account of the study being reported; that no important aspects of the study have been omitted; and that any discrepancies from the study as originally planned have been explained.

## Supporting information


Supporting File 1

Supporting File 2

Supporting File 3

## Data Availability

The datasets generated and/or analyzed during the current study are not publicly available due to privacy and confidentiality considerations, but are available from the corresponding author on reasonable request.
