# Supplementary material for: Knowledge of Blood‐Borne Infections Among Secondary School Students in Riyadh, Saudi Arabia
Source: Health Sci Rep. 2026 Sep 23;9(10):e73283. doi: 10.1002/hsr2.73283 (PMC13598678; doi:10.1002/hsr2.73283)
Supplement: Supplementary file 1 [file HSR2-9-e73283-s001.docx]

**Supplementary File 1
Complete English Translation of the Original Questionnaire**

**Knowledge of Blood-Borne Infections Among Secondary School Students in Riyadh, Saudi Arabia**

Note: The original questionnaire was administered in Arabic. This document is a complete English translation prepared for publication and preserves the original item numbering and order. Items 43–102 comprise 60 numbered entries; item 65 (“Other, please specify”) is an open-ended, non-scored field. Therefore, the knowledge scale contains 59 scored items.

# Participant Information

Questionnaire No.: ____________________

School Name: _________________________

# Part I: Personal Information

Please place a check mark (✓) next to the appropriate answer.

**1. Age:**

☐ ________ years

**2. Gender:**

☐ Male

☐ Female

**3. Nationality:**

☐ Saudi

☐ Non-Saudi

**4. Educational grade:**

☐ First secondary grade

☐ Second secondary grade

☐ Third secondary grade

**5. Educational track:**

☐ Scientific

☐ Literary/Humanities

☐ Not applicable

**6. Number of family members (including father and mother):**

☐ 1–3

☐ 4–6

☐ More than 6

**7. With whom do you live?**

☐ In the family home

☐ In a relative’s home

☐ In another place

**8. Mother’s educational level:**

☐ Illiterate

☐ Reads and writes

☐ Primary school

☐ Intermediate school

☐ Secondary school

☐ Diploma

☐ Bachelor’s degree

☐ Postgraduate studies

**9. Father’s educational level:**

☐ Illiterate

☐ Reads and writes

☐ Primary school

☐ Intermediate school

☐ Secondary school

☐ Diploma

☐ Bachelor’s degree

☐ Postgraduate studies

**10. Does your mother work?**

☐ Yes – occupation: ____________________

☐ No

☐ Deceased

☐ Retired

**11. Does your father work?**

☐ Yes – occupation: ____________________

☐ No

☐ Deceased

☐ Retired

**12. Approximate monthly family income:**

☐ Less than 3,000 SAR

☐ 3,000–6,000 SAR

☐ 6,001–9,000 SAR

☐ More than 9,000 SAR

☐ I do not know

**13. Is the monthly family income sufficient?**

☐ Yes

☐ No

# Part II: Medical History

Place a check mark (✓) next to the answer that applies to you. Please answer all questions.

## A. Have you had any of the following diseases?

**Organic diseases**

14. Hypertension	☐ Yes	☐ No

15. Diabetes mellitus	☐ Yes	☐ No

16. Tuberculosis	☐ Yes	☐ No

17. Hepatitis	☐ Yes	☐ No

18. Other, please specify: ____________________	☐ Yes	☐ No

**Psychological diseases**

19. Depression	☐ Yes	☐ No

20. Other, please specify: ____________________	☐ Yes	☐ No

**Surgical procedures**

21. Tonsillectomy	☐ Yes	☐ No

22. Gallbladder surgery	☐ Yes	☐ No

23. Other, please specify: ____________________	☐ Yes	☐ No

**Hereditary diseases**

24. Diabetes	☐ Yes	☐ No

25. Hypertension	☐ Yes	☐ No

26. Sickle cell anemia	☐ Yes	☐ No

27. Mediterranean anemia (thalassemia)	☐ Yes	☐ No

28. Hereditary bleeding disorder (hemophilia)	☐ Yes	☐ No

29. Other, please specify: ____________________	☐ Yes	☐ No

## B. Medication History

30. Do you take medication regularly? ☐ Yes	☐ No

31. If yes, please list the medications: ______________________________________________

## C. Blood-Related Practices

32. Have you previously donated blood? ☐ Yes	☐ No

33. Have you previously received a blood transfusion? ☐ Yes	☐ No

34. Have you ever had blood tests performed? ☐ Yes	☐ No

35. Other, please specify: ______________________________________________

## D. Have you previously attended a lecture/seminar on any of the following?

36. Blood-borne diseases	☐ Yes	☐ No	Location: ____________________

37. Infectious diseases	☐ Yes	☐ No	Location: ____________________

38. Sexually transmitted diseases such as syphilis and gonorrhea	☐ Yes	☐ No	Location: ____________________

39. Hepatitis	☐ Yes	☐ No	Location: ____________________

40. AIDS (acquired immunodeficiency syndrome)	☐ Yes	☐ No	Location: ____________________

41. Blood donation	☐ Yes	☐ No	Location: ____________________

42. Other, please specify: ______________________________________________

# Part III: Knowledge of Blood-Borne Diseases

For each statement below, select one response: Correct / Incorrect / I do not know. Please answer all items.

**Definition of blood-borne diseases**

43. They are diseases that are easily transmitted from one person to another. ☐ Correct	☐ Incorrect	☐ I do not know

44. They are diseases transmitted through blood transfusion. ☐ Correct	☐ Incorrect	☐ I do not know

45. They are diseases transmitted through breathing. ☐ Correct	☐ Incorrect	☐ I do not know

**Types of blood-borne diseases**

46. Hepatitis B. ☐ Correct	☐ Incorrect	☐ I do not know

47. Salmonella. ☐ Correct	☐ Incorrect	☐ I do not know

48. Hepatitis C. ☐ Correct	☐ Incorrect	☐ I do not know

49. AIDS (acquired immunodeficiency syndrome). ☐ Correct	☐ Incorrect	☐ I do not know

50. Smallpox. ☐ Correct	☐ Incorrect	☐ I do not know

**Type of microorganism causing blood-borne diseases**

51. Virus. ☐ Correct	☐ Incorrect	☐ I do not know

52. Bacteria. ☐ Correct	☐ Incorrect	☐ I do not know

53. Mosquito. ☐ Correct	☐ Incorrect	☐ I do not know

**Modes of transmission of blood-borne diseases**

54. Blood transfusion. ☐ Correct	☐ Incorrect	☐ I do not know

55. Unprotected sexual intercourse with an infected person. ☐ Correct	☐ Incorrect	☐ I do not know

56. Body fluids such as saliva and body secretions. ☐ Correct	☐ Incorrect	☐ I do not know

57. Breastfeeding from an infected mother. ☐ Correct	☐ Incorrect	☐ I do not know

58. Unsterile and contaminated equipment such as cupping instruments. ☐ Correct	☐ Incorrect	☐ I do not know

59. Open wounds (e.g., a razor cut) and scratches. ☐ Correct	☐ Incorrect	☐ I do not know

**Symptoms associated with blood-borne diseases**

60. The microorganism may remain in the body for a long period without symptoms. ☐ Correct	☐ Incorrect	☐ I do not know

61. There are no symptoms. ☐ Correct	☐ Incorrect	☐ I do not know

62. High fever. ☐ Correct	☐ Incorrect	☐ I do not know

63. Pain (abdominal pain, back pain, etc.). ☐ Correct	☐ Incorrect	☐ I do not know

64. Severe weakness and fatigue. ☐ Correct	☐ Incorrect	☐ I do not know

**65. Other, please specify: ______________________________________________**

Item 65 is an open-ended, non-scored field.

**Complications resulting from blood-borne diseases**

66. Liver fibrosis (cirrhosis). ☐ Correct	☐ Incorrect	☐ I do not know

67. Liver cancer. ☐ Correct	☐ Incorrect	☐ I do not know

68. Weakening of the body’s immunity and susceptibility to persistent infection. ☐ Correct	☐ Incorrect	☐ I do not know

69. General weakness and severe fatigue. ☐ Correct	☐ Incorrect	☐ I do not know

70. Tumors. ☐ Correct	☐ Incorrect	☐ I do not know

71. Enzyme/hormone dysfunction. ☐ Correct	☐ Incorrect	☐ I do not know

**Incorrect practices that may facilitate transmission of infection**

72. Using another person’s toothbrush/miswak. ☐ Correct	☐ Incorrect	☐ I do not know

73. Using another person’s needle/injection. ☐ Correct	☐ Incorrect	☐ I do not know

74. Sharing shaving tools between individuals. ☐ Correct	☐ Incorrect	☐ I do not know

75. Sharing cupping equipment between individuals without sterilization. ☐ Correct	☐ Incorrect	☐ I do not know

76. Sharing nail-clipping instruments without proper cleaning between individuals. ☐ Correct	☐ Incorrect	☐ I do not know

77. Using ear/body piercing instruments without sterilization. ☐ Correct	☐ Incorrect	☐ I do not know

78. Using tattooing instruments between individuals without sterilization. ☐ Correct	☐ Incorrect	☐ I do not know

79. Shaking hands with a person infected with a blood-borne disease. ☐ Correct	☐ Incorrect	☐ I do not know

80. Hugging a person infected with a blood-borne disease. ☐ Correct	☐ Incorrect	☐ I do not know

81. Kissing a person infected with a blood-borne disease. ☐ Correct	☐ Incorrect	☐ I do not know

82. Using the infected person’s eating utensils. ☐ Correct	☐ Incorrect	☐ I do not know

83. Drinking contaminated water. ☐ Correct	☐ Incorrect	☐ I do not know

**Methods of treatment for blood-borne diseases**

84. There are known treatments at the research and trial stage. ☐ Correct	☐ Incorrect	☐ I do not know

85. Herbal medicines. ☐ Correct	☐ Incorrect	☐ I do not know

86. There is no treatment. ☐ Correct	☐ Incorrect	☐ I do not know

**Methods of prevention of blood-borne diseases**

87. Routine screening and counseling for pregnant women. ☐ Correct	☐ Incorrect	☐ I do not know

88. Routine screening for family members of an infected patient. ☐ Correct	☐ Incorrect	☐ I do not know

89. Avoid sharing injection needles between individuals. ☐ Correct	☐ Incorrect	☐ I do not know

90. Wash hands thoroughly after dealing with an infected person. ☐ Correct	☐ Incorrect	☐ I do not know

91. Administer hepatitis B vaccine. ☐ Correct	☐ Incorrect	☐ I do not know

92. Administer hepatitis C vaccine. ☐ Correct	☐ Incorrect	☐ I do not know

93. Administer vaccines/immunizations when available. ☐ Correct	☐ Incorrect	☐ I do not know

94. Avoid sharing toothbrushes between individuals. ☐ Correct	☐ Incorrect	☐ I do not know

95. Avoid sharing razor blades between individuals. ☐ Correct	☐ Incorrect	☐ I do not know

96. Avoid breastfeeding by infected women. ☐ Correct	☐ Incorrect	☐ I do not know

97. Continue breastfeeding if the mother is infected. ☐ Correct	☐ Incorrect	☐ I do not know

98. Disinfect personal instruments such as scissors and nail clippers. ☐ Correct	☐ Incorrect	☐ I do not know

99. Sterilize instruments used by the dentist. ☐ Correct	☐ Incorrect	☐ I do not know

100. Use single-use instruments when performing cupping. ☐ Correct	☐ Incorrect	☐ I do not know

101. Perform a blood test to detect hepatitis (B and C) when needed or when infection is suspected. ☐ Correct	☐ Incorrect	☐ I do not know

102. Perform a blood test to detect acquired immunodeficiency virus (HIV) when needed or when infection is suspected. ☐ Correct	☐ Incorrect	☐ I do not know

**Scoring note:** Items 43–64 and 66–102 constitute the 59 scored knowledge items. Item 65 is an open-ended response and is not included in the total knowledge score.
