# Supplementary material for: Knowledge of Blood‐Borne Infections Among Secondary School Students in Riyadh, Saudi Arabia
Source: Health Sci Rep. 2026 Sep 23;9(10):e73283. doi: 10.1002/hsr2.73283 (PMC13598678; doi:10.1002/hsr2.73283)
Supplement: Supplementary file 2 [file HSR2-9-e73283-s002.docx]

**Supplementary Table S2. Associations between previous exposure to health education and knowledge level (N = 686)**

| **Health education topic** | **Previous exposure** | **n** | **Poor knowledge** **n (row %)** | **Moderate knowledge** **n (row %)** | **Good knowledge** **n (row %)** | **χ²** | **p value** | **Holm-adjusted** **p value** | **Cramer’s V** |
| --- | --- | --- | --- | --- | --- | --- | --- | --- | --- |
| Any previous health education | No | 408 | 288 (70.6) | 82 (20.1) | 38 (9.3) | 21.103 | <.001 | — | 0.175 |
|  | Yes | 278 | 149 (53.6) | 83 (29.9) | 46 (16.5) |  |  |  |  |
| Blood-borne infections | No | 567 | 379 (66.8) | 129 (22.8) | 59 (10.4) | 16.391 | <.001 | <.001 | 0.155 |
|  | Yes | 119 | 58 (48.7) | 36 (30.3) | 25 (21.0) |  |  |  |  |
| Infectious diseases | No | 546 | 368 (67.4) | 123 (22.5) | 55 (10.1) | 18.630 | <.001 | <.001 | 0.165 |
|  | Yes | 140 | 69 (49.3) | 42 (30.0) | 29 (20.7) |  |  |  |  |
| Sexually transmitted diseases | No | 594 | 399 (67.2) | 138 (23.2) | 57 (9.6) | 34.987 | <.001 | <.001 | 0.226 |
|  | Yes | 92 | 38 (41.3) | 27 (29.3) | 27 (29.3) |  |  |  |  |
| Hepatitis | No | 627 | 413 (65.9) | 142 (22.6) | 72 (11.5) | 14.809 | <.001 | .0012 | 0.147 |
|  | Yes | 59 | 24 (40.7) | 23 (39.0) | 12 (20.3) |  |  |  |  |
| HIV/AIDS | No | 523 | 355 (67.9) | 118 (22.6) | 50 (9.6) | 21.011 | <.001 | <.001 | 0.175 |
|  | Yes | 163 | 82 (50.3) | 47 (28.8) | 34 (20.9) |  |  |  |  |
| Blood donation/transfusion | No | 550 | 367 (66.7) | 123 (22.4) | 60 (10.9) | 11.317 | .0035 | .0035 | 0.128 |
|  | Yes | 136 | 70 (51.5) | 42 (30.9) | 24 (17.6) |  |  |  |  |

**Note.** Pearson’s chi-square tests were used. The composite variable was coded as yes when a participant reported exposure to at least one of the six listed health education topics. Holm adjustment was applied to the six topic-specific comparisons only; the composite comparison was not included in the adjustment. All expected cell counts exceeded 5. Statistical significance was set at p < .05.
