# Supplementary material for: Knowledge of Blood‐Borne Infections Among Secondary School Students in Riyadh, Saudi Arabia
Source: Health Sci Rep. 2026 Sep 23;9(10):e73283. doi: 10.1002/hsr2.73283 (PMC13598678; doi:10.1002/hsr2.73283)
Supplement: Supplementary file 3 [file HSR2-9-e73283-s003.docx]

**Supplementary Table S1. Item-level results for the 59 scored knowledge items (N = 686)**

| **Domain** | **Item** | **Knowledge item** | **Correct response** | **Correct, n (%)** |
| --- | --- | --- | --- | --- |
| Definitions and examples | 43 | Blood-borne diseases transfer easily from one person to another. | True | 123 (17.93) |
| Definitions and examples | 44 | Blood-borne diseases are transmitted through blood. | True | 580 (84.55) |
| Definitions and examples | 45 | Blood-borne diseases are transmitted through breathing. | False | 416 (60.64) |
| Definitions and examples | 46 | Hepatitis B. | True | 171 (24.93) |
| Definitions and examples | 47 | Salmonella. | False | 103 (15.01) |
| Definitions and examples | 48 | Hepatitis C. | True | 146 (21.28) |
| Definitions and examples | 49 | HIV/AIDS. | True | 418 (60.93) |
| Definitions and examples | 50 | Smallpox. | False | 275 (40.09) |
| Microbe type | 51 | Viruses. | True | 389 (56.71) |
| Microbe type | 52 | Bacteria. | True | 221 (32.22) |
| Microbe type | 53 | Mosquitoes. | False | 150 (21.87) |
| Modes of transmission | 54 | Blood transfusion. | True | 550 (80.17) |
| Modes of transmission | 55 | Unprotected sexual contact with an infected person. | True | 469 (68.37) |
| Modes of transmission | 56 | Body fluids (e.g., saliva and body secretions). | True | 156 (22.74) |
| Modes of transmission | 57 | Breastfeeding by an infected mother. | True | 197 (28.72) |
| Modes of transmission | 58 | Poorly ventilated environments. | False | 301 (43.88) |
| Modes of transmission | 59 | Open wounds and scratches. | True | 451 (65.74) |
| Signs and symptoms | 60 | The pathogen may remain in the body for years without symptoms. | True | 180 (26.24) |
| Signs and symptoms | 61 | Blood-borne infections have no symptoms. | False | 372 (54.23) |
| Signs and symptoms | 62 | High fever. | True | 284 (41.40) |
| Signs and symptoms | 63 | Abdominal or back pain. | True | 230 (33.53) |
| Signs and symptoms | 64 | Excessive fatigue and weakness. | True | 341 (49.71) |
| Complications | 66 | Liver cirrhosis/fibrosis. | True | 240 (34.99) |
| Complications | 67 | Liver cancer. | True | 211 (30.76) |
| Complications | 68 | Low immunity and recurrent infection. | True | 462 (67.35) |
| Complications | 69 | General fatigue and extreme weakness. | True | 335 (48.83) |
| Complications | 70 | Tumors. | True | 215 (31.34) |
| Complications | 71 | Enzyme/hormonal imbalance. | True | 246 (35.86) |
| Incorrect practices | 72 | Sharing toothbrushes or siwak. | True | 403 (58.75) |
| Incorrect practices | 73 | Sharing needles or injections. | True | 590 (86.01) |
| Incorrect practices | 74 | Sharing shaving tools. | True | 455 (66.33) |
| Incorrect practices | 75 | Sharing cupping instruments without disinfection. | True | 522 (76.09) |
| Incorrect practices | 76 | Sharing nail-cleaning or nail-cutting tools without disinfection. | True | 296 (43.15) |
| Incorrect practices | 77 | Sharing piercing instruments without sterilization. | True | 432 (62.97) |
| Incorrect practices | 78 | Sharing tattoo needles without sterilization. | True | 427 (62.24) |
| Incorrect practices | 79 | Shaking hands with an infected person. | False | 401 (58.45) |
| Incorrect practices | 80 | Hugging an infected person. | False | 389 (56.71) |
| Incorrect practices | 81 | Kissing an infected person. | False | 279 (40.67) |
| Incorrect practices | 82 | Sharing eating utensils with an infected person. | False | 170 (24.78) |
| Incorrect practices | 83 | Drinking contaminated water. | False | 113 (16.47) |
| Treatment | 84 | Treatment is available based on research and clinical evidence. | True | 437 (63.70) |
| Treatment | 85 | Herbal remedies are an appropriate treatment. | False | 150 (21.87) |
| Treatment | 86 | No treatment is available. | False | 319 (46.50) |
| Prevention | 87 | Routine testing for pregnant women. | True | 479 (69.83) |
| Prevention | 88 | Routine testing for patients' family members. | True | 530 (77.26) |
| Prevention | 89 | Avoid sharing needles. | True | 560 (81.63) |
| Prevention | 90 | Wash hands after contact with an infected person. | True | 395 (57.58) |
| Prevention | 91 | Receive hepatitis B vaccination. | True | 384 (55.98) |
| Prevention | 92 | Receive hepatitis C vaccination. | False | 38 (5.54) |
| Prevention | 93 | Receive recommended vaccinations when available. | True | 484 (70.55) |
| Prevention | 94 | Avoid sharing toothbrushes. | True | 444 (64.72) |
| Prevention | 95 | Avoid sharing shaving blades or razors. | True | 485 (70.70) |
| Prevention | 96 | Infected women should stop breastfeeding. | True | 327 (47.67) |
| Prevention | 97 | Infected women can continue breastfeeding. | False | 266 (38.78) |
| Prevention | 98 | Disinfect personal tools such as scissors and nail cutters. | True | 484 (70.55) |
| Prevention | 99 | Sterilize dental instruments. | True | 508 (74.05) |
| Prevention | 100 | Use single-use cupping instruments. | True | 404 (58.89) |
| Prevention | 101 | Test for hepatitis B and C when infection is suspected. | True | 444 (64.72) |
| Prevention | 102 | Test for HIV when infection is suspected. | True | 492 (71.72) |

**Note.** Percentages were calculated using N = 686 as the denominator. Item 65 ("Other, please specify") was open-ended and was excluded from scoring. Correct responses reflect the original study scoring key. Items addressing body fluids and breastfeeding should be interpreted cautiously because transmission risk and breastfeeding recommendations vary according to the specific blood-borne infection.
